# The Interaction Between Phonological and Semantic Processing in Reading Chinese Characters

**DOI:** 10.3389/fpsyg.2018.02748

**Published:** 2019-01-10

**Authors:** Min Dang, Rui Zhang, Xiaojuan Wang, Jianfeng Yang

**Affiliations:** Key Laboratory of Behavior and Cognitive Neuroscience in Shaanxi Province, School of Psychology, Shaanxi Normal University, Xi’an, China

**Keywords:** word reading, Chinese character, phonological, semantic, interaction

## Abstract

The contemporary models of visual word recognition reached a consensus on a cooperative division of labor between phonological and semantic processing. We examine how reading is influenced by the interaction of two processing in Chinese character reading since the ideographic property of the Chinese writing system is perfectly suitable to address this issue. The current study investigated whether Chinese character reading requires the interaction between orthography-to-phonology consistency and semantic processing of the whole character (imageability, Experiment 1) or the semantic radical (transparency, Experiment 2). Experiment 1 showed a significant effect of the consistency and a marginal effect of the imageability, but no interaction between the two. Experiment 2 found a significant effect of the semantic transparency and its interaction with phonological consistency, where the transparent effect was significant for inconsistent characters but not for consistent ones. The current finding provided direct evidence of the interplay between phonological and semantic processing and shed light on the language-general reading model.

## Introduction

A general agreement of the contemporary reading models (Hillis and Caramazza, 1995; Seidenberg, 2011) is that there exists an interaction between phonological and semantic processing involved in the computation from print to sound. The cooperative division of labor between these two processing has been implemented in a connectionist model to simulate normal and disordered development of reading Chinese characters (Yang et al., 2013). However, no study reported the direct evidence of phonological and semantic interaction in reading Chinese characters.

The view of the cooperative division of labor between phonological and semantic processing mainly received evidence from studies in alphabetic languages. The interaction was a distinctive prediction of the connectionist triangle model (Seidenberg and McClelland, 1989) compared to the Dual Route Cascaded reading model (Coltheart et al., 2001). Its robust evidence from human adults was the observation showing a semantic imageability effect only for irregular words, not for regular words (Strain et al., 1995, 2002; Strain and Herdman, 1999). This behavioral phenomenon has been simulated and explained by the cooperative division of labor between phonological and semantic pathways in a computational model (Harm and Seidenberg, 2004). Subsequent neural evidence supporting this computational model has been found, where fMRI study observed a trade-off between brain regions of phonological and semantic processing (Frost et al., 2005). Further evidence has been shown more recently using fMRI studies with the multiparametric analytic approach (Graves et al., 2010) and the dynamic causal models (Boukrina and Graves, 2013).

Chinese character reading is perfectly suitable to address the interplay between phonological and semantic processing. In Chinese, more than 80% of characters are phonograms (Li and Kang, 1993) which consist of a phonetic radical and a semantic radical. The phonetic radical provides phonological cues for the character’s pronunciation, and the semantic radical indicates the semantic category of the character’s meaning. Unlike English where words have an arbitrary mapping between spelling and meaning, Chinese character has a systematic mapping from orthographic components to their meaning. The previous studies demonstrated that Chinese character reading is influenced by both phonetic radicals (Lee et al., 2005; Williams and Bever, 2010) and semantic radicals (Feldman and Siok, 1999; Zhou et al., 2012).

Some studies have shown that both the phonetic and semantic radicals involved in the processing of Chinese characters. On one hand, both the sound and the meaning of phonetic radicals involved in the processing of Chinese characters. The pronunciation of phonetic radical influences Chinese character reading by showing regularity and consistency effect. The regularity effect is that a regular character with a similar pronunciation to its phonetic radical was recognized faster and more accurate than an irregular one (Hue, 1992; Peng and Yang, 1997; Williams and Bever, 2010). The consistency effect is that a consistent character with a similar pronunciation to all characters in the same phonetic family was recognized faster and more accurate than an inconsistent one (Fang et al., 1986; Jared, 1997; Lee et al., 2005). The meaning of phonetic radical engaged in the processing of Chinese characters by showing a semantic priming effect in character reading. For example, in a primed naming task, Zhou and Marslen-Wilson (1999) found that the priming character (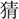,” cai[1], guess) facilitated the target (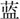,” lan[2], blue) because its phonetic radical (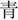,” qing[1], blue/green) was semantically relevant to the target. The engagement of the phonetic radical’s meaning also received neural evidence from an ERP study (Lee et al., 2006).

On the other hand, the semantic radical showed its phonological and semantic impact on Chinese character reading. For example, a transparent character, whose meaning is relevant to the meaning of its semantic radical, was processed faster and more accurate than an opaque one (unrelated) in a semantic categorization task (Williams and Bever, 2010). In addition to the semantic role, the phonological information of semantic radicals also engaged in and affected the character processing. Zhou et al. (2000) found a priming effect that the target (e.g., 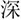,” shen[1], deep) was primed by the prime (e.g., 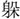,” duo[3], hide) since the prime’ s semantic radical (e.g., 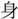,” shen[1], body) shared the sound with the target. In a recent study, Zhou et al. (2012) used opaque phonograms as primes to examine the role of the semantic radicals. They found the meaning of semantic radical (e.g., 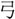,” gong[1], bow) of the prime (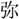,” mi[2], full) can facilitate the naming of target characters (e.g., 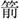,” jian[4], arrow) which were semantically related to the semantic radical of the prime. They also found the pronunciation of the semantic radical (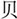,” bei[4], shell) of the prime (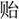,” yi[2], present) can facilitate the naming of targets (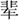,” bei[4], generation) which were homophone of the semantic radical. The above findings suggested that the semantic radical can provide both the phonological and semantic cues for the character’s naming.

Although both the phonetic and semantic radicals involved in Chinese character recognition (Wang et al., 2016a, 2017), there is no direct empirical study showing the interaction between phonological and semantic processing. Previous studies have demonstrated a possible interaction existed in Chinese character reading. In a survey of disordered reading, Bi et al. (2007) reported a dysgraphic patient who had a left temporal lobe ischemic damage resulting in dementia. Despite the patient produced semantic errors in both comprehension and production task, he could read characters he wholly or partially understood. However, for the characters whose meaning he did not know, the patient was unable to read and showed a regularity effect. The result indicated that the patient’s performance relies on both phonological and semantic processing. When semantic information was not available, the patient read characters mainly by relying on phonological information to show a regularity effect that the regular characters were read more accurately than irregular ones.

The interaction has also received indirect evidence from a case study of reading development. Shu et al. (2005) reported three children showing two different types of dyslexia: Child L was semantic dyslexia and had semantic deficits, but he had intact phonological awareness, so his reading mainly depended on phonological processing and showed a regularity effect. On the contrary, Child J and Q were phonological dyslexia, who had the normal semantic ability but an impaired phonological awareness, so their reading accuracy was low and did not show regularity effect. The results of dyslexic children suggested that reading development depends on the cooperative labor of phonological and semantic ability.

Furthermore, connectionist modeling has provided an algorithmic explanation for the interaction between phonological and semantic processing. Using the same model architecture and algorithm broadly implemented in English model, Yang et al. (2009) developed a connectionist model of Chinese character reading. The trained model successfully simulated the regularity and consistency effects as well as their interaction with the frequency which was a landmark finding in normal adult reading. Further impairment during the training of the model has simulated two types of developmental dyslexia reported in Shu et al. (2005). When a decay happened in the semantic pathway, the model simulated the reading pattern of the surface dyslexic Child L. In contrast, the model with a decayed phonological pathway has simulated the reading pattern of the phonological dyslexic Child J and Q (Yang et al., 2013). That is, the cooperative division of labor between phonological and semantic processing have been adapted and explained by the computational algorithm for Chinese character reading.

Moreover, recent cognitive neuroscience studies have suggested an interactive network between the phonological and semantic neural circuits for Chinese character reading. Phonological processing mapped the activities from the left ventral Occipital-Temporal gyrus (vOT) to the left frontal gyrus (e.g., inferior frontal gyrus, insular) (Tan et al., 2005; Wu et al., 2012). The semantic route mapped the activities from the vOT to the posterior temporal gyrus (e.g., Middle Temporal Gyrus and Angular Gyrus) (Wu et al., 2012; Wang et al., 2016b). The cooperative labor between two routes formed a shared neural network involved in reading real, pseudo-characters as well as character-like stimuli (Wang et al., 2011; Yang et al., 2012). For instance, using a multiple parametric correlation approach, Wang et al. (2016b) found that the activation of phonologically related brain regions (left middle frontal gyrus, inferior frontal gyrus, and insula) enhanced with increasing phonological cues embodied in radicals. Correspondently, the semantically related regions (left angular gyrus, middle temporal gyrus) received more activation with more semantic cues embodied in the radicals. The results offered potential neural correlates of the interaction between phonological and semantic processing in Chinese character reading.

In sum, previous studies have shown indirect evidence for the interaction between phonological and semantic processing in reading Chinese characters. To date, previous studies have failed to demonstrate any convincing evidence. In a recent study, Wang et al. (2017) simultaneously manipulated the semantic transparency and phonetic regularity in a lexical decision task. They only found a significant main effect of transparency. Their main effect of regularity and its interaction with semantic transparency did not reach significance.

The current study aims to directly investigate whether Chinese character reading required the interaction between phonological processing and semantic processing at character (Experiment 1) or radical level (Experiment 2). Experiment 1 intended to replicate the finding in English studies by manipulating the phonetic consistency and semantic imageability. Since the mapping from spelling to meaning in English is relative arbitrary, previous studies manipulated the semantic factor at the whole word level, e.g., concreteness, imageability. Experiment 1 aimed to examine whether the word-level imageability also engaged in reading Chinese characters and interplayed with phonetic consistency. At the same time, Chinese characters have a systematic mapping from spelling to meaning so that the sub-lexical semantic cues involve in reading (Feldman and Siok, 1999; Williams and Bever, 2010). Experiment 2 manipulated semantic factor at sub-lexical level (radical semantic transparency) to examine the interaction between the phonetic consistency and the semantic transparency. Considering the distinctive properties of Chinese characters from English, we predicted an interaction between phonological and semantic effects in Experiment 2, no matter the interaction in Experiment 1 is observed or not.

## Experiment 1

### Methods

#### Participants

Thirty university students (16 females, aged 18–27) from Shaanxi Normal University took part in the experiment. All participants were native speakers of Mandarin Chinese with normal or corrected-to-normal vision, with no history of neurological disease or learning disability. They provided written informed consent and were paid a stipend.

#### Materials and Design

The selection of materials consisted of two stages: database screening and subjective rating phase. At the database screening phase, 1708 regular phonograms were first picked up from the total 4468 characters in a Chinese corpus (Language Instruction Institute of Beijing Language College, 1986). All picked items were filtered by the consistency level (Shu et al., 2003; Yang et al., 2009) resulting in two group characters: 1017 high consistent (level ≥ 0.8), and 253 low consistent (level ≤ 0.5) characters. Each group was further filtered by the frequency (≤80/million) and the phonetic family size (≥3). Consequently, the database screening phase produced 1023 candidate items including 794 high and 229 low consistent characters.

At the subjective rating phase, all candidate items were firstly browsed by the experimenter to conduct a preliminary screening of the semantic imageability. The initial meaning evaluation removed items with ambiguous meaning or polysemous characters based on the subjective definition of the meaning. Two hundred items survived at the first screening. The number of high- and low-imageable characters for each group were enough and roughly equal: 107 for high and 93 for the low consistent group. Then, those items were rated by 21 college students in a 7 points scale to evaluate whether the meaning of the character is imageable (1 for the lowest and 7 for the highest imageable meaning).

Based on the average score of rating results, a set of 120 characters was picked up as the final experimental items with 30 characters in each cell created by crossing consistency level (high/low) with imageability (high/low). As shown in Table 1, four conditions have matched value of frequency [*F*(3,116) = 0.000, *p* = 1.000], the number of phonetic radical neighbors [*F*(3,116) = 1.20, *p* = 0.314], the number of radicals [*F*(3,116) = 2.06, *p* = 0.109], and the number of strokes [*F*(3,116) = 1.85, *p* = 0.142]. As for consistency level, the difference between high and low consistent characters was significant both for high [*t*(29) = 28.67, *p* < 0.001] and low imageable characters [*t*(29) = 29.52, *p* < 0.001]. The consistency level was matched between high and low imageable characters both for high [*t*(29) = 0.10, *p* = 0.925], and for low [*t*(29) = 0.39, *p* = 0.703] consistent characters. As for imageability rating score, the difference between high and low imageability characters were significant both for high [*t*(29) = 31.42, *p* < 0.001] and low consistent characters [*t*(29) = 33.52, *p* < 0.001]. The rate score of imageabilty was match between high and low consistent items for high [*t*(29) = 1.15, *p* = 0.259] and for low [*t*(29) = 0.64, *p* = 0.525] imageable items.

**Table 1:** The matching results of materials in Experiment 1.

	High consistency		Low consistency	
	High IMG	Low IMG	High IMG	Low IMG
Consistency level	0.97 (0.03)	0.97 (0.04)	0.23 (0.14)	0.22 (0.13)
IMG rate	6.13 (0.42)	2.17 (0.64)	6.00 (0.46)	2.08 (0.41)
Frequency	20.52 (17.00)	20.57 (22.81)	20.48 (18.43)	20.41 (23.82)
Families	8.36 (3.33)	8.60 (3.59)	9.90 (4.14)	9.80 (4.49)
Strokes	11.73 (3.27)	10.03 (3.13)	11.30(2.90)	10.43 (3.03)
Radicals	3.70 (1.35)	3.20 (0.75)	3.13 (0.72)	3.27 (0.89)


*IMG, imageability; out of brackets are the means, in brackets are the standard deviations.*

#### Procedure

Participants sat in a comfortable distance from the screen (about 60 cm) and were instructed to read aloud single character into a microphone as quickly and accurately as possible. On each trial, a fixation cross appeared for 500 ms, after which the screen was cleared for 120 ms, and a single character was presented for up to 2000 ms (or until a response was made). Stimuli were presented centrally, in white against a black background using the 28 pt Songti font. Stimulus presentation and response latency collection was controlled using E-Prime 2.0 software. Stimuli were presented in a different, randomized order for each participant. All stimuli were presented in 2 runs, and each run was about 5 min. The task lasted for about 10 min.

### Results

**FIGURE 1:**
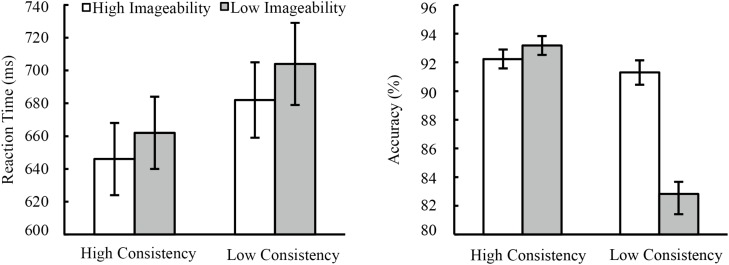
The result of Experiment 1. The RTs **(Left)** showed main effects of phonological consistency and semantic imageability but no interaction between them. The naming accuracy **(Right)** showed two main effects and their interaction.

All participants’ data were included for analysis, and the average naming accuracy was 90.50%. The data of error responses were defined as the reaction time (RT) of incorrect naming or the RT beyond the range of 150–1500 ms. The extreme data were the RTs beyond 2.5SD from the mean RT of each condition. All error or extreme data were replaced by the mean RT in each condition for each participant. The averaged RT and naming accuracy was shown in Figure 1.

For the reaction time, 2 (consistent level: high/low) × 2 (imageability: high/low) ANOVA analysis showed a significant main effect of the consistency level [*F*_1_(1, 29) = 59.63, *p* < 0.001, η^2^ = 0.673; *F*_2_(1, 116) = 12.05, *p* < 0.005, η^2^ = 0.094]. Participants named faster for high consistent than for low consistent characters. The main effect of imageability also reached significant for subject analysis [*F*_1_(1,29) = 12.81, *p* < 0.005, η^2^ = 0.306], but not for item analysis [*F*_2_(1,116) = 2.43, *p* = 0.122, η^2^ = 0.02]. The interaction between the consistency level and the imageability was not significant [*F*_1_(1,29) = 0.34, *p* = 0.567, η^2^ = 0.011; *F*_2_(1,116) = 0.18, *p* = 0.674, η^2^ = 0.002].

The same ANOVA was conducted for the naming accuracy. The main effect of consistency level was significant [*F*_1_(1,29) = 42.39, *p* < 0.001, η^2^ = 0.588; *F*_2_(1,116) = 4.18, *p* < 0.05, η^2^ = 0.036]. The main effect of imageability also reached significant for subject analysis (*F*_1_(1,29) = 17.3, *p* < 0.001, η^2^ = 0.357), but not for item analysis [*F*_2_(1,116) = 1.99, *p* = 0.161, η^2^ = 0.017]. An interesting finding was the significant consistency x imageability interaction observed for subject analysis [*F*_1_(1,29) = 26.03, *p* < 0.001, η^2^ = 0.5], not for items analysis [*F*_2_(1,116) = 2.29, *p* = 0.133, η^2^ = 0.02]. The simple effect showed more accurate naming for high than low imageable characters when reading low consistent characters [*F*_1_(1, 29) = 28.29, *p* < 0.001, η^2^ = 0.5], but the simple imageability effect didn’t reach significant in naming high consistent characters [*F*_1_(1, 29) = 0.05, *p* = 0.832, η^2^ = 0.000].

However, ANOVA results only showed significant imageability effect for subject analysis, not for item analysis. To validate our results, we conducted a linear mixed-effects model (Baayen et al., 2008) both for RT and ACC analysis. The model included fixed effects of the consistency (high/low) and the imageability (high/low), random effects for subjects and items. The RT results showed a significant main effect of the consistency (β = 38.92, *t* = 3.69, *p* < 0.001), a marginally significant effect of imageability (β = 18.38, *t* = 1.74, *p* = 0.084), and non-significant interaction between consistency and imageability (β = 6.27, *t* = 0.30, *p* = 0.767). The ACC results only showed a significant effect of consistency (β = –0.06, *t* = –2.04, *p* < 0.05). The main effect of imageability (β = –0.04, *t* = –1.43, *p* = 0.157) and its interaction with consistency was not significant (β = –0.09, *t* = –1.50, *p* = 0.136).

### Discussion

Experiment 1 examined the interaction between phonological processing and lexical-semantic processing. Results showed a significant main effect of phonetic consistency, a marginal effect of meaning imageability and no interaction between them.

The consistency effect is a typical phenomenon in visual word recognition that indicates an influence of phonological processing. It is a common effect reported in the studies of alphabetic languages (Andrews, 1982; Jared, 1997, 2002), in which high consistent words are read faster and more accurate than those low consistent words. The consistency effect has been reported in Chinese character reading both for adults (Fang et al., 1986; Lee et al., 2005; Yang et al., 2009) and children (Yang and Peng, 1997; Xing et al., 2004). It interacted with the lexical frequency in behavioral (Hue, 1992) and computational modeling studies (Yang et al., 2009; Chang et al., 2016). Experiment 1 replicated the findings in previous studies and showed a faster and more accurate naming for high than low consistent characters suggesting general phonological processing in word reading for non-alphabetic languages.

However, there might be a different mechanism underlying the consistency effect in reading Chinese characters. In alphabetic languages, such as English, the orthography-phonology mapping is mainly the transform from grapheme to phoneme. The consistency means the systematic mapping from the spelling to its sound at the phoneme/rime level. Whereas Chinese characters have no systematic mapping from grapheme to phoneme, the consistency means the pronunciation of a phonogram is influenced by other members’ pronunciations in the same phonetic family. This language difference explained why the neural basis of the consistency effect were different in English and Chinese studies. The English studies mainly found the activation in left posterior Superior Temporal Gyrus (pSTG) for LOW > HIGH consistent words (Binder et al., 2005a; Bolger et al., 2008). The engagement of the left pSTG suggested the spelling-to-sound computing underlying the consistency effect. On the contrary, the consistency effect in Chinese mainly activated the left inferior frontal gyrus (Lee et al., 2004, 2010) for LOW > HIGH consistent characters. The IFG was the unique region significantly correlated with the consistency level for character reading using a multi-parametric approach (Wang et al., 2016b). Because of the weak spelling-to-sound mapping in Chinese, the left IFG engaged in reading inconsistent characters to resolve the ambiguity of phonological representations.

Another finding in Experiment 1 is the marginal effect of the imageability. The imageability has been manipulated to examine the contribution of semantic processing in English word reading (Strain et al., 1995; Woollams, 2005). High imageable words were recognized faster and more accurate than low imageable ones (Binder et al., 2005b), and correspondently evoked more activation at semantic related brain regions, such as the bilateral Angular Gyrus (Binder et al., 2009; Graves et al., 2010). The imageability effect was significant especially for items with low frequent and inconsistent orthography-phonology mapping (Strain et al., 1995; Woollams, 2005). Despite ample findings in alphabetic languages, the imageability effect in Chinese character reading is lack. To our knowledge, Chinese researchers have considered the imageability as a variable of the character’s meaning in a corpus naming norms (Liu et al., 2007). Our finding in Experiment 1 is the first time to reveal the imageability effect in Chinese character reading directly.

However, Experiment 1 did not found the interaction between consistency and imageability effect. The result is different from previous findings of English word reading. Strain et al. (2002) showed a robust interaction between imageability and consistency in reading English words. The interaction has also received evidence in an fMRI study (Frost et al., 2005). In our data, Experiment 1 did not observe the interaction, which may result from the ideographic properties of the Chinese writing system. For Chinese characters, semantic radical can provide reliable cues of the meaning. Thus the lexical semantic factor (e.g., imageability) is relatively weak. To further address this possibility, Experiment 2 manipulated the semantic radical as the cue of semantic processing to further investigate its interaction with phonological processing in reading Chinese characters.

## Experiment 2

### Methods

#### Participants

Thirty university students (15 females, aged 17–21) were recruited from Shaanxi Normal University. All participants were native speakers of Mandarin Chinese with normal or corrected-to-normal vision, with no history of neurological disease or learning disability. They provided written informed consent and were paid a stipend.

#### Materials and Design

Same as the Experiment 1, the selection of materials consisted of two phases: database screening and subjective rating phase. At the database screening phase, all regular phonograms were first picked up from the Chinese corpus (Language Instruction Institute of Beijing Language College, 1986). Two group items were identified according to the high (level ≥ 0.8) and low (level ≤ 0.5) consistency level (Shu et al., 2003; Yang et al., 2009). Each group was further filtered by the frequency (≤80/million) and the family size (≥3) to produce the candidate items.

At the subjective rating phase, all candidate items were firstly browsed by the experimenter. The initial semantic rating removed items with ambiguous meaning or polysemous characters based on the subjective definition of the meaning. One hundred eighty-eight items survived after the preliminary screening of the relationship between the meaning of the character and its semantic radical. This step remained an enough and roughly equal number of the high- and low-transparent characters for each group: 106 for high and 82 for low consistent characters. Then, the 188 candidate items were rated by 20 college students in a 7 points scale (1 for the opaque and 7 for the transparent meaning) to evaluate how the semantic radical indicates the whole character’ meaning.

A set of 120 characters were picked up as the final experimental items with 30 characters in each cell created by crossing consistency level (high/low) with meaning transparency (transparent/opaque). As shown in Table 2, four conditions have matched value of frequency [*F*(3,116) = 0.09, *p* = 0.964], the number of phonetic radical neighbors [*F*(3,116) = 2.10, *p* = 0.105], the number of radicals [*F*(3,116) = 1.32, *p* = 0.271], and the number of strokes [*F*(3,116) = 1.73, *p* = 0.165].

**Table 2:** The matching results of materials in Experiment 2.

	High consistency		Low consistency	
	**Transparent**	**Opaque**	**Transparent**	**Opaque**
Consistency	0.99 (0.03)	0.97 (0.06)	0.29 (0.13)	0.27 (0.15)
Transparency	6.23 (0.43)	2.40 (0.60)	6.22 (0.49)	2.63 (0.75)
Frequency	19.81 (15.30)	19.94 (18.57)	19.72 (11.42)	17.96 (20.00)
Families	8.13 (3.46)	8.00(4.52)	9.70 (4.58)	10.37 (4.71)
Strokes	10.87 (2.75)	9.70(2.57)	10.80(3.04)	11.27 (2.62)
Radicals	3.37(1.02)	3.00 (0.89)	3.07 (0.96)	3.40 (0.95)


The phonetic consistency level was matched between transparent and opaque characters. The difference between high and low consistent characters was significant both for transparent [*t*(29) = 29.05, *p* < 0.001] and opaque characters [*t*(29) = 20.91, *p* < 0.001]. There was no difference between transparent and opaque characters neither for high [*t*(29) = 1.35, *p* = 0.189], nor for low [*t*(29) = 0.55, *p* = 0.588] consistent items. As for transparency rating score, the difference between transparent and opaque characters were significant both for high [*t*(29) = 32.30, *p* < 0.001] and low consistent characters [*t*(29) = 19.00, *p* < 0.001]. But, the rating score was matched between high and low consistent characters both for transparent [*t*(29) = 0.10, *p* = 0.921], and for opaque items [*t*(29) = –1.11, *p* = 0.276].

#### Procedure

Same to Experiment 1.

### Results

**FIGURE 2:**
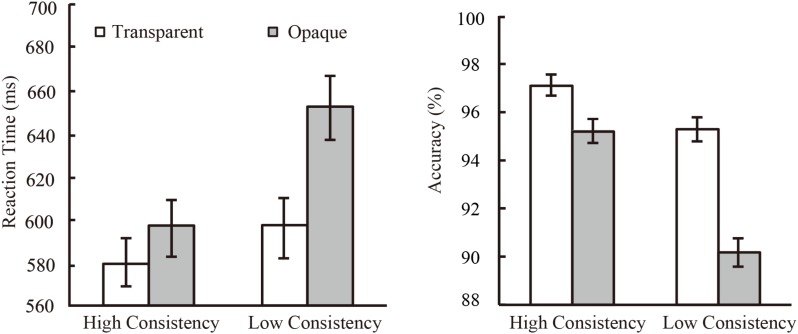
The result of Experiment 2. Both the naming RTs **(Left)** and accuracy **(Right)** showed significant effects of phonological consistency, semantic transparency, and their interaction.

All participants completed the naming task, and the average accuracy was 94.3%. The method of raw data analysis was the same as Experiment 1. The averaged RT and naming accuracy was shown in Figure 2.

For the reaction time, the 2(consistent level: high/low) × 2(transparency: transparent/opaque) ANOVA analysis showed a significant main effect of the consistency level [*F_1_*(1,29) = 93.15, *p* < 0.001, η^2^ = 0.763; *F*_2_(1,116) = 15.56, *p* < 0.001, η^2^ = 0.118]. Same to the Experiment 1, participants named faster for high consistent than for low consistent characters. The main effect of transparency also reached significant both for subject analysis and item analysis [*F*_1_(1,29) = 94.14, *p* < 0.001, η^2^ = 0.764; *F*_2_(1,116) = 14.69, *p* < 0.001, η^2^ = 0.112]. The transparent characters were named faster than the opaque characters indicating a facilitating effect of the radical meaning in character naming. More importantly, the result showed a significant consistency x transparency interaction [*F*_1_(1,29) = 56.27, *p* < 0.001, η^2^ = 0.660; *F*_2_(1,116) = 4.53, *p* < 0.05, η^2^ = 0.038] because the transparent characters were named faster than the opaque ones for low consistent condition [*F*_1_(1,29) = 127.87, *p* < 0.001, η^2^ = 0.815; *F*_2_(1,117) = 15.81, *p* < 0.001, η^2^ = 0.119], but there only was difference between them for high consistent characters for item analysis [*F*_1_(1,29) = 18.57, *p* < 0.001, η^2^ = 0.390; *F*_2_(1,117) = 1.29, *p* = 0.258, η^2^ = 0.011].

For the naming accuracy, the ANOVA showed a significant main effect of consistency [*F*_1_(1,29) = 33.58, *p* < 0.001, η^2^ = 0.571; *F*_2_(1,116) = 4.37, *p* < 0.05, η^2^ = 0.038] and a significant main effect of transparency for subject analysis [*F*_1_(1,29) = 33.11, *p* < 0.001, η^2^ = 0.5], a marginally significance for item analysis [*F*_2_(1,116) = 3.37, *p* = 0.069, η^2^ = 0.029]. A significant consistency × transparency interaction was also observed for subject analysis [*F*_1_(1,29) = 13.79, *p* < 0.005, η^2^ = 0.333], however, the interaction did not reach significant for item analysis [*F*_2_(1,116) = 1.15, *p* = 0.287, η^2^ = 0.01]. The transparent characters were named more accurate than the opaque ones for low consistent characters both for subject and item analysis [*F*_1_(1,29) = 36.37, *p* < 0.001, η^2^ = 0.571; *F*_2_(1, 117) = 4.1, *p* < 0.05, η^2^ = 0.037], but a significant transparent effect for high consistent characters only for subject analysis [*F*_1_(1,29) = 4.63, *p* < 0.05, η^2^ = 0.138; *F*_2_(1,117) = 0.28, *p* = 0.594, η^2^ = 0.000].

We also conducted a linear mixed-effects model (Baayen et al., 2008) that included fixed effects of the consistency (high/low) and transparency (transparent/opaque), random effects for subjects and items. RT Results showed significant main effects of the consistency (β = 36.20, *t* = 3.87, *p* < 0.001) and the transparency (β = 36.35, *t* = 3.89, *p* < 0.001). Moreover, the consistency and the transparency have a significant interaction (β = 37.44, *t* = 2.00, *p* < 0.05). The simple effect found the transparent effect on low consistent condition, that is, the naming speed of transparent characters are faster than opaque characters (β = 55.07, χ^2^ = 11.54, *p* < 0.001). While, no transparent effect was found on the high consistent condition (β = 17.63, χ^2^ = 2.90, *p* = 0.088). ACC results showed a significant effect of the consistency (β = –0.04, *t* = –2.10, *p* < 0.05), a marginally significant effect of the transparency (β = –0.03, *t* = –1.84, *p* = 0.069). The interaction between two fixed factors was not significant (β = –0.04, *t* = –1.05, *p* = 0.296).

To exclude the potential influence of imageability on the results of Experiment 2, we performed a linear mixed effects model (Baayen et al., 2008) for RT analysis, including fixed effects of consistency (high/low), transparency (transparency/opaque), imageability (high/low), and random effects for subjects and items. We used a maximal random effect structure (Barr et al., 2013): for items, slopes for all of the predictors (consistency, transparency, and imageability) and their interactions; for subjects, a random intercept. Results showed significant main effects of consistency (β = 47.07, *t* = 4.15, *p* < 0.005), transparency (β = 38.60, *t* = 3.41, *p* < 0.01), and a marginally significant interaction between consistency and transparency (β = 46.59, *t* = 2.06, *p* = 0.064). There was no significant main effect of imageability (β = –5.00, *t* = –0.44, *p* = 0.668), nor its interaction with others (consistency, β = –15.81, *t* = –0.70, *p* = 0.500; transparency, β = 37.04, *t* = 1.64, *p* = 0.131; and their interaction, β = –46.63, *t* = –1.03, *p* = 0.326). Thus, the Experiment 2 showed a reliable finding of consistency, transparency and their interaction.

### Discussion

Experiment 2 examined the interaction between the consistency and the transparency effect. Same to the result of Experiment 1, Experiment 2 revealed the main effects of consistency and semantic transparency. More interesting, Experiment 2 showed a robust interaction between two factors suggesting cooperation of phonological and semantic processing in Chinese character reading.

Previous studies have shown the involvement of the semantic radical in Chinese character reading. The properties of the semantic radical can be automatically activated and further influenced the character processing. In an ERPs study, Wang et al. (2016a) observed a frequency effect of the semantic radical that the high frequent radical evoked a smaller N400 than the low frequent one. The semantic radical also influenced the character reading by showing a neighborhood size and category consistency effects under radical priming paradigm (Zhang and Zhang, 2017), in which the effects were consistent with behavioral and eye movement experiments (Wang and Zhang, 2016). Moreover, the meaning of an independent semantic radical has a long-term priming effect in lexical decision task than a dependent radical (Zhang and Zhang, 2016). So that, the semantic radical can provide reliable clues and can be potential to facilitate the recognition and processing of the character.

However, no direct evidence showed an effect of semantic radicals in the naming task. Most of the semantic radical effect mainly came from indirect observation using particular paradigms (such as priming or semantically related tasks). For example, in a primed character decision task, a target phonogram was significantly facilitated by a prime that shared the target’s semantic radical and was semantically related (Feldman and Siok, 1999). When the meaning of the semantic radical is related to the character, participants’ responses were facilitated, whereas their responses were inhibited when the semantic radical is opaque to the meaning of the character (Williams and Bever, 2010). In Experiment 2, our result showed a significant transparency effect in the naming task that the character with a related meaning to its semantic radical was named faster and more accurately. The result provides direct evidence of the engagement of semantic radicals in character reading.

An important finding in Experiment 2 was the interaction between the consistency and the transparency effect. The result is the first time to reveal an interaction between phonological and semantic processing in Chinese character reading. Our result in Experiment 1 did not replicate the interaction between the consistency and imageability effect that has reported both in behavioral (Strain et al., 1995, 2002) and neuroimaging (Frost et al., 2005) studies in English word reading. However, in Experiment 2, the result successfully showed a significant interaction between the consistency and the transparency effect.

A possible interpretation of the different interaction in our two experiments may be the differential orthography-to-semantics (O-S) mapping across English and Chinese writing system. Since the arbitrary O-S mapping for English words, the imageability effect was mainly manipulated as a semantic factor and was further shown its interaction with phonological processing in previous studies of English word reading (Strain et al., 1995, 2002). The imageability is a lexical factor that depends on the successful access of an entire word’s meaning. On the contrast, in Chinese, the meaning of a phonogram can be partially accessed from the cues of the semantic radical. Reading the character relies more on the meaning of its radicals rather than itself. Corresponds, the interaction between phonological and semantic processing depends on the meaning of the radicals. Thus, experiment 1 only found a main effect of the imageability but failed on its interaction with the phonological factor. Experiment 2 showed significant effects both for the semantic transparency and its interaction with the consistency.

## General Discussion

The primary purpose of the current study is to examine the interaction between phonological processing and semantic processing both at the character (imageability) and the radical level (transparency). Experiment 1 found the main effects of the imageability and the consistency, without the interaction between them. Experiment 2 found a significant effect of semantic transparent and its interaction with the phonological consistency. The finding is the first time to provide the direct evidence for the interaction between phonological and semantic processing in a naming task of Chinese character reading.

The most important finding in our study is the interaction between semantic transparency and phonetic consistency effect in Experiment 2. The finding is in line with the previous studies in English word reading. In a series of studies, Strain et al. (1995) found a robust interaction between the regularity and the imageability for low-frequency words. They observed a significant imageability effect for irregular words but not for regular words. Their results showed that, when phonological information was insufficient for irregular words, participants could use the semantic information to perform the naming task and show the imageability effect. Their finding was supported by the findings in an fMRI study showing a trade-off effect between the phonological and semantic associated brain regions (Frost et al., 2005). That is, the high imageable words reduced the activation in inferior frontal gyrus which was associated with phonological processing, but increased the activation in the posterior part of the middle temporal gyri and angular gyri which were semantically related regions. The cooperative labor of phonological and semantic neural circuits was supported in subsequent fMRI studies using the multiparametric analysis approach (Graves et al., 2010) and the dynamic causal models (Boukrina and Graves, 2013).

Our findings provided direct evidence for the contribution of the semantic radical in reading Chinese characters. The view of the interaction between the phonology and the semantics has been proposed in computational modeling of Chinese (Yang et al., 2013), case studies of patients with brain injury (Bi et al., 2007), and brain imaging studies (Wang et al., 2016b). However, the view was still at the theoretical level, and the direct empirical evidence in the naming task was a lack. On the one hand, although previous studies have revealed that the semantic radical engaged in character recognition, no study showed the transparency effect of the relationship between the meaning of the semantic radical and its character in a naming task. On the other hand, no direct evidence reported the interaction between the phonology and the semantic factors in characters reading aloud. The current study is the first time to show the semantic transparency effect and its interaction with the phonological consistency effect.

The current study has an important implication for the model of reading. Although both the Dual Route Cascaded (DRC) and the connectionist approach agreed that it exists the combination of sub-lexical and lexical route, the DRC model holds the view that the semantic system is beyond the scope of the reading model and not suitable for Asian languages including Chinese characters (Coltheart et al., 2001). The connectionism approach proposed that word reading in different languages share a universal interaction between phonological and semantic processing. In Chinese, the connectionist computational model has successfully simulated the reading phenomenon of healthy adults (Yang et al., 2009), sub-types of developmental dyslexia (Yang et al., 2013). These results indirectly indicated a division of labor between phonological and semantic processing. Here, our finding offered direct empirical evidence of the interaction.

Moreover, our present results are in line with findings of recent fMRI studies of Chinese character reading. Increasing evidence has shown a shared brain network for processing of different types of characters, such as real-, pseudo-characters, and artificial (Wang et al., 2011; Yang et al., 2012). The neural network for different types of characters reflected the dynamic cooperation of phonological and semantic processing neural routes (Wang et al., 2016b). Taking account of the cooperative labor between two neural routes summarized in alphabetic studies (Carreiras et al., 2014), the interaction between two routes might be the general cognitive and neural mechanism of visual word recognition across languages.

However, one limitation of the current study is that we did not match the initial phoneme of the stimuli. Onset effects are a potential issue in collecting naming data, and It has been shown to account for a great deal of variance in naming data (Balota et al., 2004). To exclude the possible influence of VOT (voice onset time), we did a *post hoc* analysis and found no difference between long vs. short VOT syllables both for RT and ACC. The long/short VOT were defined according to the acoustic features in Mandarin Chinese. Long VOT syllables began with a voiced phoneme (e.g., p/t/k/c/ch/q) and short VOT syllables began with an unvoiced phoneme (e.g., b/d/g/z/zh/j). In Experiment 1, there were 18 long and 42 short VOT syllables out of the 120 items. The difference between long and short syllables was not significant neither for RT [*t*(58) = –0.59, *p* = 0.558] nor for ACC [*t*(58) = 0.13, *p* = 0.892]. In Experiment 2, there were 22 long and 33 short VOT syllables. The difference between long and short syllables was not significant for RT [*t*(53) = –1.29, *p* = 0.203], but marginal significant for ACC [*t*(53) = 1.95, *p* = 0.064]. To remove the potential impact entirely, further study should match the initial phonemes better.

Another limitation was the underlying mechanism of the interaction between phonological and semantic processing. Our study is the first step to confirm the interaction in reading Chinese characters. It is not clear why such interaction was only for two sub-lexical processing, but not for lexical semantic and sub-lexical phonological processing. The underlying cognitive processing and neural basis is an open question in future studies.

## Conclusion

In this study, we used the imageability of whole characters and the transparency of semantic radicals to determine the cooperative labor of phonological and semantic processing in Chinese characters reading. At the radical level, we found the interaction between the semantic transparency and the phonological consistency. From a cross-language perspective, the result provides direct evidence for the cooperative division of labor between phonological and semantic processing in word reading. In line with previous indirect evidence from acquired (Bi et al., 2007) and developmental dyslexia (Shu et al., 2005), computational modeling (Yang et al., 2009, 2013), and neuroscience studies (Wang et al., 2016b; Zhao et al., 2017), the current study indicated a universal connectionist model of visual word recognition across languages.

## Author Contributions

MD, RZ, XW, and JY designed the research. MD and RZ collected the data. MD, RZ, and JY analyzed the data. XW and JY interpreted the data. MD, XW, and JY wrote the manuscript.

## Conflict of Interest Statement

The authors declare that the research was conducted in the absence of any commercial or financial relationships that could be construed as a potential conflict of interest.
